# Precision Medicine in Childhood Cancer: The Influence of Genetic Polymorphisms on Vincristine-Induced Peripheral Neuropathy

**DOI:** 10.3390/ijms25168797

**Published:** 2024-08-13

**Authors:** Luciana Marangoni-Iglecias, Susana Rojo-Tolosa, Noelia Márquez-Pete, Yasmín Cura, Noelia Moreno-Toro, Cristina Membrive-Jiménez, Almudena Sánchez-Martin, Cristina Pérez-Ramírez, Alberto Jiménez-Morales

**Affiliations:** 1 Clinical Analysis Laboratory Unit, Hospital Universitário Maria Aparecida Pedrossian HUMAP-UFMS, Av. Sen. Filinto Müler, 355, Vila Ipiranga, Campo Grande 79080-190, Brazil; luciana.iglecias@gmail.com; 2 Pharmacogenetics Unit, Pharmacy Service, University Hospital Virgen de las Nieves, Avda. de las Fuerzas Armadas 2, 18004 Granada, Spainyacura@uc.cl (Y.C.); cristina.membrive95@gmail.com (C.M.-J.); almudena.sanchez.martin.sspa@juntadeandalucia.es (A.S.-M.); cperezramirez87@ugr.es (C.P.-R.); alberto.jimenez.morales.sspa@juntadeandalucia.es (A.J.-M.); 3 Biosanitary Research Institute ibs.GRANADA, Avda. de Madrid 15, 18012 Granada, Spain; 4 Department of Biochemistry and Molecular Biology II, Faculty of Pharmacy, Campus Universitario de Cartuja, University of Granada, 18011 Granada, Spain; 5 Pneumology Service, University Hospital Virgen de las Nieves, Avda. de las Fuerzas Armadas 2, 18004 Granada, Spain; 6 Pediatric Service, Santa Ana Regional Hospital, Av. Enrique Martín Cuevas, s/n, Motril, 18600 Granada, Spain; noeliamoreno.t@gmail.com

**Keywords:** vincristine, peripheral neuropathy, pediatric oncology, pharmacogenetics, precision medicine, toxicity

## Abstract

Cancer is the leading cause of disease-related death among children. Vincristine (VCR), a key component of childhood cancer treatment protocols, is associated with the risk of peripheral neuropathy (PN), a condition that may be reversible upon drug discontinuation but can also leave lasting sequelae. Single nucleotide polymorphism (SNP) in genes involved in VCR pharmacokinetics and pharmacodynamics have been investigated in relation to an increased risk of PN. However, the results of these studies have been inconsistent. A retrospective cohort study was conducted to investigate the potential association of drug transporter genes from the ATP-binding cassette (*ABC)* family and the centrosomal protein 72 (*CEP72*) gene with the development of PN in 88 Caucasian children diagnosed with cancer and treated with VCR. Genotyping was performed using real-time PCR techniques for the following SNPs: *ABCB1* rs1128503, *ABCC1* rs246240, *ABCC2* rs717620, and *CEP72* rs924607. The results indicated that age at diagnosis (OR = 1.33; 95% CI = 1.07–1.75) and the *ABCC1* rs246240 G allele (OR = 12.48; 95% CI = 2.26–100.42) were associated with vincristine-induced peripheral neuropathy (VIPN). No association was found between this toxicity and *CEP72* rs924607. Our study provides insights that may contribute to optimizing childhood cancer therapy in the future by predicting the risk of VIPN

## 1. Introduction

Childhood cancer has an incidence of 15 to 20 cases per 100,000 children and is the leading cause of disease-related death in children globally [1]. Children and young people cancer treatment involves particular requirements and concerns due to the ongoing growth and development of the patients. Children are more vulnerable to the severe side effects of cancer therapies compared to adults due to the increased susceptibility of their developing organs [2]. Over the past five decades, there have been significant advances in the diagnosis and treatment of many types of childhood cancer, resulting in a reduction in mortality rates. In most high-income countries, overall survival is over 80% [3]. Treatment for childhood cancer often involves patient participation in multicenter clinical trials. The specific treatment plan may include chemotherapy, radiotherapy, and surgery, depending on the diagnosis type, severity, and tumor location [4]. Nonetheless, efforts continue to improve treatment safety due to the narrow therapeutic index of the drugs used in order to optimize benefits while minimizing the danger of life-threatening toxicities. With the increasing success in treating cancers, it is now important to focus on preventing and minimizing the adverse effects of treatment, both in the short and long term [5]. Even though the dosages for anti-leukemic drugs are determined based on defined protocols, accurately predicting toxicity remains challenging due to significant variations across individuals [6].

Vincristine (VCR) is a member of the vinca alkaloid group found naturally in the plant *Madagascar periwinkle Catharanthus roseus* and has been employed for the therapeutic management of various cancers since 1963 [7]. It functions as a cytostatic drug by attaching to the β-tubulin subunit of αβ-tubulin heterodimers, disrupting the assembly of the mitotic spindle cell in metaphase, which in turn, blocks cell division and results in limited growth of the tumor cells. However, its biological effect lacks specificity [2,8]. Currently, VCR is commonly included in treatment protocols for childhood cancers such as acute lymphocytic leukemia (ALL), Hodgkin and non-Hodgkin lymphoma, sarcomas, neuroblastoma, and Wilms tumor, and off-label uses include the treatment of Ewing sarcoma and meduloblastoma [9,10]. VCR clinical use is hampered by the onset of neurotoxicity, particularly peripheral neuropathy (PN), which often requires dose reduction or treatment discontinuation [11,12,13]. Vincristine-induced peripheral neuropathy (VIPN) manifests as peripheral nerve damage, resulting in a range of symptoms, including sensory impairments (sensory/tactile dysfunction, limb numbness, and tingling), motor deficits (paresthesia, impaired balance, and abnormal gait), and autonomic neuropathy (dysuria, sexual dysfunction, and ileus paralysis). Furthermore, VCR can induce auditory and visual impairments [14,15,16]. Approximately 80% of VCR-treated patients experience short or long-term VIPN, with elderly patients at higher risk of developing movement disorders, and of these, approximately 30% may experience severe symptoms [5,10,17,18]. Signs and symptoms of VIPN may manifest within a week of initiating treatment and might last for up to 12 months after reducing the dose [12]. In most cases, VCR-induced neurotoxicity resolves upon discontinuation of treatment, although some patients report persistent symptoms in the long term [19]. The incidence of VIPN may differ among various populations [17]. Although there are still conflicting results, prior research has identified that some sociodemographic characteristics of the populations studied can influence the occurrence of VIPN; other important factors are those connected to the drug, such as the frequency of administration, total accumulated dose, and interaction with concomitant medications, as well as the individual characteristics of the patient [20]. Individuals of white race and older age exhibit a higher susceptibility to VIPN. Furthermore, people with Caucasian ethnicity appear to be at a higher risk [10]. In addition to the factors described above, single nucleotide polymorphisms (SNPs) have been related to the higher risk for VIPN [11,12]. Unfortunately, there is a lack of knowledge about biomarkers that can predict the increased risk of developing VIPN, and currently, no therapeutic interventions are available to prevent or reduce the incidence of it in cancer patients [2,21]. 

Although various instruments have been employed to evaluate and quantify VIPN in children undergoing cancer treatment, diagnosis of PN primarily depends on patient self-reporting, presenting particular challenges in pediatric patients due to potential difficulties in effectively communicating their symptoms [22]. The Common Terminology Criteria for Adverse Events (CTCAE) [23] is widely used to assess the severity of adverse events like VIPN, but it demonstrates limited sensitivity in detecting motor and sensory neuropathy, especially in mild or severe cases. Electrodiagnostic testing is another technique used to evaluate VIPN; nevertheless, this approach is more intrusive, painful, and agonizing, making it unsuitable for regular VIPN evaluation. Other tools that combine questionnaires and standardized physical examinations have been developed for use in children [5,12,22].

The antineoplastic agent VCR exerts its action by interfering with the formation of microtubules in the mitotic spindle during metaphase [24]. It has been confirmed that genetic factors can influence the development of VIPN by modifying the rate of VCR elimination or elevating susceptibility [22]. The centrosomal protein 72 (*CEP72)* gene encodes the microtubule-organizing centrosomal protein 72, which plays a role in the formation of microtubules during cell division [25]. The *CEP72* rs924607 polymorphism has been associated with reduced expression of the *CEP72* gene, leading to increased microtubule instability. This microtubule destabilization is precisely the mechanism of action of VCR [5]. VCR is metabolized in the liver and excreted primarily in feces. The transport and elimination of the drug are mediated by membrane transport proteins, including members of the ATP-binding cassette (ABC) transporter superfamily. Therefore, the expression and functionality of these proteins can significantly impact drug clearance and, consequently, its toxicity profile. The ABC membrane transporters, such as ABCB1, ABCC1, and ABCC2, have a significant impact on the transport of VCR in cells. ABCB1 and ABCC2 are involved in eliminating VCR through bile, while ABCC1 helps transport VCR into the bloodstream. Changes in the activity of these carrier proteins could explain the differences in VCR toxicity among patients [26]. Several studies have examined the potential role of SNPs in drug transporter genes (*ABC*) and genes involved in nerve cell function (*CEP72*) on the risk of developing VIPN, yet the results have been inconclusive [11,24,25,27,28].

Predicting which patients may develop VIPN continues to be a challenging task. Pharmacogenomics can help to elucidate an individual’s predisposition to experiencing side effects [10]. To date, there are no genetic biomarkers with sufficient evidence to identify which patients are prone to VIPN. Therefore, this study aimed to investigate the influence of SNPs in *ABC* family transporter genes (*ABCB1* rs1128503, *ABCC1* rs246240, and *ABCC2* rs717620) and the *CEP72* gene (rs924607) on the risk of VIPN in a Caucasian pediatric population diagnosed with cancer.

## 2. Results

### 2.1. Sociodemographic and Clinicopathologic Characteristics

The study included a total of 88 Caucasian pediatric patients with ages between 1 and 18 years treated with VCR between 2005 and 2022. All the patients were concurrently receiving at least one other concomitant medication according to the diagnosed disease and the treatment protocol (anthracycline, asparaginase, glucocorticoid, mercaptopurine, methotrexate etoposide, cyclophosphamide). The clinicopathologic and sociodemographic data are shown in Table 1. The median age at diagnosis was 6 (3–9) years; 46/88 (52.27%) were male, and 45/88 (51.14%) had a family history of cancer. The patients were diagnosed with ALL (49/88; 55.68%), non-Hodgkin lymphoma (15/88; 17.05%), Ewing sarcoma (7/88; 7.95%), Hodgkin lymphoma (6/88; 6.82%), ependymoma (4/88; 4.55%), and other cancers (7/88; 7.95%). Neurotoxicity was documented in the medical records of 10/88 (11.36%) patients, with PN reported in 8/88 (9.09%) patients. 

### 2.2. Genotype Distribution

**Figure 1:**
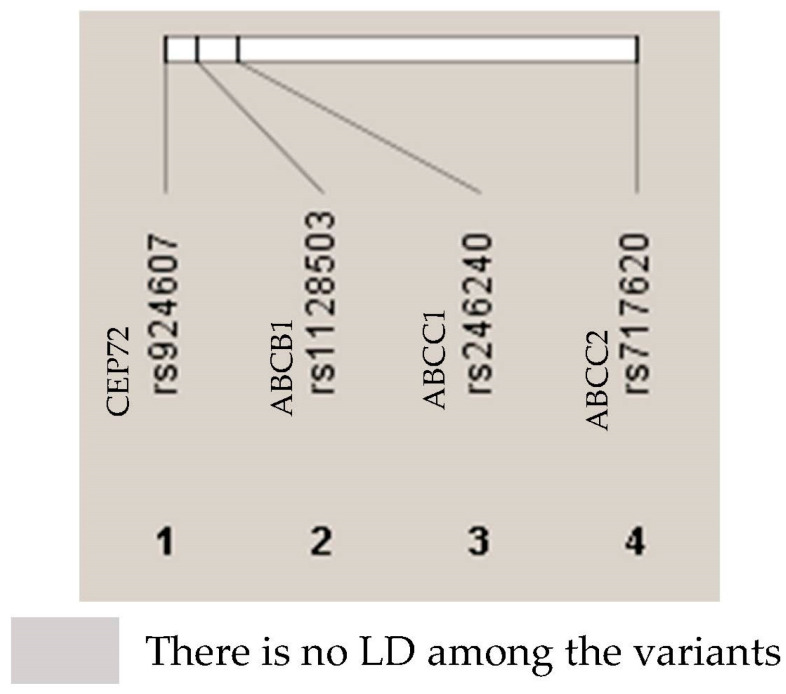
Linkage disequilibrium (LD).

Genotyping was successful for all patients and SNPs included. The distribution of the studied SNPs followed the Hardy–Weinberg equilibrium (HWE) model between the expected and observed heterozygosity (Table S1). No evidence of linkage disequilibrium (LD) was observed among the SNPs (Figure 1). All of the SNPs exhibited a minor allele frequency (MAF) > 15%, and none were excluded from the study (Table S2). 

### 2.3. Influence of Clinicopathologic and Genetic Variables on Peripheral Neuropathy in Pediatric Cancer Patients Treated with Vincristine

In bivariate analysis, age at diagnosis was associated with the incidence of PN in any degree (OR = 1.22; 95% CI = 1.03–1.50; *p* = 0.029) (Table 2). Additionally, a trend was observed between Hodgkin lymphoma and PN (OR = 15.33; 95% CI = 2.11–126.49; *p* = 0.051; ALL vs. Hodgkin lymphoma) (Table 2). The genetic variants associated with PN were the *ABCC1* rs246240—AG/GG genotypes (OR = 5.78; 95% CI = 1.16–32.03; *p* = 0.004; and OR = Inf; 95% CI = 2.37–NA; *p* = 0.004; GG vs. AA), and the *ABCC1* rs246240—G allele (OR = 7.00; 95% CI = 1.21–50.19; *p* = 0.013; G vs. AA) (Table 3). No association was found between the remaining sociodemographic, clinicopathologic, and genetic variables and PN.

Multivariate analysis confirmed that the independent variables associated with VIPN to any degree in pediatric cancer patients were age at diagnosis (OR = 1.33; 95% CI = 1.07–1.75; *p* = 0.016) and the *ABCC1* rs246240—G allele (OR = 12.45; 95% CI = 2.26–100.42; *p* = 0.006; G vs. AA) (Table 4). These associations continued to be significant after multiple comparison adjustments were applied (Table 4).

## 3. Discussion

The antineoplastic agent VCR is included in numerous treatment protocols for pediatric cancer patients. VCR is a cytotoxic drug that specifically acts on the β-tubulin subunit of αβ-tubulin heterodimers that form the centrosome’s microtubules. It disrupts the structure of mitotic spindles and prevents cancer cells from dividing by causing the depolymerization of microtubules [2]. In addition to their importance in cell division, microtubules are essential for cell shape maintenance and the movement of organelles and vesicles. Microtubules are dynamic and can polymerize and depolymerize quickly, allowing the cell to reorganize its cytoskeleton in response to different stimuli [29]. Despite its usefulness as a cytostatic agent, VCR is associated with an increased risk of PN in the short and long term [11,17]. VIPN can manifest with motor, sensory, and autonomic symptoms of varying intensities, making diagnosis in children challenging due to their difficulty in reporting symptoms [22]. While VIPN may resolve after drug withdrawal, it can also result in persisting sequelae [24]. The pathophysiology of VIPN remains unclear. However, some authors suggest that the primary mechanism for VIPN is the capacity of VCR to disrupt axonal transport by impairing the myelination of nerve fibers due to its strong attraction towards axonal microtubules [7]. In addition to this significant effect, VCR can stimulate immune cells, such as microglia, resulting in neuroinflammation. Furthermore, VCR can alter calcium metabolism at the mitochondrial level. Together, these additional pathways contribute to the development of VIPN [12,30]. Various authors have identified age, ethnicity, sex, drug dose, and SNPs as potential risk factors [10,31,32]. Our findings suggest that both age at diagnosis and the SNP *ABCC1* rs246240 are associated with an increased risk of VIPN in pediatric cancer patients (*p* = 0.016 and *p* = 0.006, respectively; Table 4).

The association between age and VIPN has been extensively investigated and has yielded contradictory results. Several studies have produced inconsistent findings regarding the metabolism of VCR in children compared to adults. Some studies suggest that children metabolize VCR more quickly than adults, which could imply greater exposure to the drug’s toxic effects over a shorter period. On the other hand, other studies have not found a direct relationship between age and VCR pharmacokinetics, suggesting that other factors might be influencing PN. In a systematic review published in 2016 by Hersman et al., a significant association was found between an increased risk of PN and older age in a multi-ethnic adult population (Asian, Pacific Islander, and Black) treated with taxanes (*p* = 0.030; OR = 1.04; 95% CI = 1.01–1.07) [33]. However, in 2017, another systematic review by Velde et al., covering studies in children of multiple ethnicities (Black, White, Hispanic, Asian, African, American, and Caucasian) with cancer diagnosis and treated with VCR, could not determine if age was related to the risk of PN as the reviewed studies were either contradictory or did not find an association [22]. Biological characteristics of the patient such as age could explain part of this variability. In children, the immature development of the peripheral nervous system and differences in neuronal regeneration capacity could contribute to increased susceptibility to VCR-induced neuropathy. Additionally, the lower capacity of children to metabolize and excrete VCR could lead to greater accumulation of the drug in nerve tissues, exacerbating the risk of PN. In contrast, in adults, age-related changes in liver and kidney function might affect VCR pharmacokinetics differently, altering the toxicity profile and the incidence of PN.

Drug disposition (absorption, distribution, and excretion), clinical efficacy, and toxicity depend on transporters, which mediate substrate and drug entry and efflux [34]. The ABC transporters family comprises a group of protein carriers that utilize ATP hydrolysis to move their substrates through the cell membrane [35]. The *ABCC1* gene is located on chromosome 16 and encodes the multidrug resistance-associated protein 1 (MRP1) [36]. MRP1 is a membrane transporter protein present in the blood–brain barrier that plays a role in the excretion of endogenous substances and antineoplastic drugs [37]. Consequently, it has been demonstrated to influence individual susceptibility to drug toxicity. The impact of MRP1 in drug excretion has been corroborated by animal experimentation, which has revealed increased chemosensitivity in subjects deficient in this transporter protein [38]. There is scarce information regarding the influence of this SNP on VIPN. The results of our study revealed an association between the *ABCC1* rs246240 G allele and an elevated risk of VIPN (*p* = 0.006, Table 2). Our findings are consistent with those of Franca et al. in a study conducted on a population of 508 primarily European Caucasian children with ALL. The study reported that the GG genotype of the *ABCC1* rs246240 polymorphism was associated with an elevated risk of neurotoxicity in patients treated with antineoplastic agents, including VCR (*p* = 0.035) [30]. The precise molecular function of the rs246240 variant in *ABCC1* remains unknown. However, Franca et al. postulated that this SNP may result in increased pump activity. This could potentially result in elevated drug concentrations in the brain, due to the ABCC1 transporter being located on the basal surface of the capillary endothelium of the blood–brain barrier [39]. Biodistribution studies in mice have shown that *ABCC1* expression and function differ between the brain and liver. This facilitates the accumulation of ABCC1 substrates in the brain, while drugs are rapidly removed from peripheral tissues [38].

The *CEP72* gene, located on chromosome 5, encodes CEP72, which is crucial for centrosome formation [38]. Numerous studies have reported that the *CEP72* rs924607 polymorphism may be implicated in an increased risk of VIPN [10,25,28,40]. In 2015, Diouf and collaborators in a genome-wide study involving two different populations of American children of different ethnicities (European, African, Asian, Hispanic and others) diagnosed with ALL and treated with VCR (n = 222 and 99, respectively) reported a significant association between the TT genotype of the *CEP72* rs924607 and the VIPN post-consolidation phase (*p* < 0.001; 95% CI = 43.9–77.6). They demonstrated that patients with the *CEP72 rs924607* polymorphism required lower mRNA levels to produce the CEP72 protein, which was confirmed in different types of cells. Carriers of the T allele of this SNP had a greater facility for connecting the regulatory protein NKX-6.3, which in turn acts as a repressor of the expression of the *CEP72* gene. Thus, the amount of mRNA produced by *CEP72* is reduced, which reduces protein levels. Reduced CEP72 levels in human neurons and leukemic cells make them more sensitive to VCR [28]. Furthermore, in 2017, a study of 49 adults of diverse ethnic backgrounds (Hispanic, Indian, White, and unknown) also identified an association between the *CEP72* rs924607 and VIPN (*p* = 0.020; OR = 4.33; 95% CI = 1.19–23) [40]. In 2019, the Canadian Pharmacogenomics Network for Drug Safety, a multicenter consortium dedicated to active surveillance and pharmacogenomics, published a report indicating that the *CEP72* rs924607 polymorphism elevates sensitivity to VCR by influencing microtubule stability, thus corroborating the association between *CEP72* rs924607 and VIPN [25]. Moreover, a systematic review and meta-analysis published in 2022 identified an association between *CEP72* rs924607 and VIPN in a population comprising patients of Black and White ethnicity, the majority of whom had been diagnosed with ALL [10]. Our study did not identify an association between the *CEP72* rs924607 polymorphism and VIPN. In accordance with our findings, in 2016, a study by Gutierrez-Camino, which included a cohort of 142 children of European origin from Spain, did not confirm the association between the *CEP72* rs924607 polymorphism and VIPN in a study conducted during the induction phase of ALL treatment [27]. This discrepancy in results between previous studies may be attributed to variations in VCR doses between different treatment protocols for ALL and to differential mechanisms involved in VIPN during the initial and final phases of treatment. This uniformity could reduce variations in VCR exposure and, consequently, in the occurrence of neuropathy. On the other hand, differences in study population and methodology could influence the frequency and expression of the studied SNP, such that our results are only consistent with the Gutierrez-Camino study, which was conducted in a population and with a sample size similar to ours. Finally, another possible cause for the lack of association in our study and the Gutierrez-Camino study could be due to statistical analysis and study power, as the statistical analyses and sample size might have limited the ability to detect an association if the effect size of the *CEP72* rs924607 polymorphism is small.

The present study provides information on the influence of age at diagnosis and one SNP in a gene encoding a transporter protein on the risk of VIPN in childhood cancer patients. This investigation was limited by its retrospective nature, with the information obtained depending on the quality of the available medical records, and by the wide range of diagnoses in the study population. However, the sample size and the fact that all the patients were treated in a single hospital with the same protocols established for their respective diagnoses, contribute to the homogeneity and quality of the research. Uniformity in treatment and follow-up can minimize confounding variables, which allowed us to more clearly assess the influence of SNPs in VIPN. In other studies, variability in treatment protocols, doses administered or differences in patient management may introduce additional factors that affect the results, and this may explain some of the differences observed between our study and the results of other studies.

Pharmacogenetics play a crucial role in the treatment of childhood cancers, particularly in managing the toxicity associated with chemotherapy. The application of pharmacogenetic analysis in clinical practice could help predict the risk of VIPN in the pediatric cancer population and may contribute to discovering the etiology of this toxicity. Further research is required to reinforce existing knowledge and identify novel genetic biomarkers in VCR transporters and targets. This could aid in predicting the risk of VIPN in pediatric cancer treatment, enabling personalized dosing and regimen adjustments, reducing the risk of severe adverse reactions, and improving overall outcomes.

## 4. Materials and Methods

### 4.1. Study Design

A retrospective observational cohort study was conducted.

### 4.2. Study Population

A total of 88 Caucasian cancer patients, aged between 2 and 14 years at the time of their cancer diagnosis, were included in the study. The patients were treated with VCR in accordance with the treatment protocol selected based on clinical criteria at the Virgen de las Nieves University Hospital (HUVN) in Granada, Spain, between 2005 and 2022.

### 4.3. Ethics Statements

The study was approved by the Ethics and Research Committee of Granada (code: 0388-M1-19) and conducted in accordance with the Helsinki Declaration. Legal guardians of the patients who participated in the study provided written consent for the collection of genetic samples, as well as for their donation to the Biobank of the Andalusian Health System. Samples were identified by alphanumeric codes.

### 4.4. Sociodemographic and Clinicopathologic Data

Sociodemographic and clinicopathologic data were collected by reviewing patient medical records using the *Diraya Estación Clínica* software version 4.10.1. The collected sociodemographic and clinicopathologic data included date of diagnosis, sex, cancer diagnosis, age at diagnosis, family history of cancer, and concurrent medications. Neurotoxicity and PN were recorded within a period of four weeks from the last cycle of VCR or at the beginning of the next phase of treatment, whichever occurred first. Toxicities were recorded as present or absent and graded according to the National Cancer Institute—Common Terminology Criteria for Adverse Events (NCI-CTCAE) version 5.0 [23] and subsequently classified as I–II for mild toxicity and III–IV for severe toxicity. In the event of multiple episodes of toxicity, the most severe grade reported was considered.

### 4.5. Genetic Variables

#### 4.5.1. DNA Isolation

Saliva samples were collected via oral swabs. Genomic DNA was extracted using the QlAamp DNA Mini Kit (Qiagen GmbH, Hilden, Germany), following the manufacturer’s instructions for purifying DNA, and was stored at −40 °C. The concentration and purity of the extracted DNA were determined using a NanoDrop^TM^ 2000 UV spectrophotometer, measuring 260/280 and 260/230 absorbance ratios (NanoDrop^TM^ Technologies Inc., Wilmington, DE, USA).

#### 4.5.2. Detection of Gene Polymorphisms

SNPs in genes encoding ABC transporters from subfamilies B and C and in the *CEP72* gene with a MAF > 15% in the European population, according to the dbSNP database from the National Institutes of Health [41], were selected. These SNPs have been previously identified as predictors of toxicity in the literature [5,11,17,25,28,39] and in the PharmGKB database [42].

SNPs were determined by real-time polymerase chain reaction (PCR) allelic discrimination assay using TaqMan™ probes (ABI Applied Biosystems, Waltham, MA, USA, QuantStudio 3 Real-Time PCR System, 96 wells) in accordance with the manufacturer’s instructions (Table 5). This procedure is based on oligonucleotide probes that are labeled with a fluorescent reporter and a quencher. When the probe does not hybridize to its target sequence, the two components are tightly coupled, preventing amplification and the generation of a fluorescence signal. Upon hybridization, conformational alterations occur in the reporter and quencher, enabling the 5′–3′ exonuclease activity of Taq polymerase to cleave this bond, thereby releasing the fluorescence emitted by the reporter and its capture by the device. Each allele is labelled with a distinct fluorochrome (VIC/FAM), thereby enabling the determination of the genotype based on the fluorescence captured by the device.

The criteria for SNP quality control were as follows:

(1) Rate of missing genotypes per SNP < 0.05.
(2) MAF > 0.01.
(3) *p*-value > 0.05 in the HWE test.

### 4.6. Statistical Analysis

Normality was assessed using the Kolmogorov–Smirnov test. Qualitative variables were presented as frequencies and percentages, whereas quantitative variables were presented as medians and percentiles (p25–p75) due to their non-normal distribution. Bivariate analysis was conducted using Fisher’s exact test and Pearson’s chi-squared test for qualitative variables, and the Mann–Whitney U test for non-parametric quantitative variables. In the SNP association analysis, models were defined as dominant ((DD, Dd) vs. dd), recessive (DD vs. (Dd, dd)), and genotypic (DD vs. dd and Dd vs. dd). Significant associations identified in the bivariate analysis underwent univariate logistic regression to calculate adjusted odds ratios (OR) and 95% confidence intervals (CI) for potential toxicity predictive factors. Variables that were significant in the bivariate analysis were further analyzed using multivariate logistic regression with a backward stepwise method. All statistical tests were two-sided, with a significance level of *p* < 0.05. Correction for false discovery rate (FDR) was applied to adjust *p*-values for multiple comparisons, with a q-value of 0.05. The genetic analyses included HWE, MAF, and LD. The LD analysis was performed using Lewontin’s D prime (D’) and LD coefficient (r^2^), and LD plots were generated using the Haploview 4.2 software [43]. Statistical analyses were conducted using the software PLINK version 1.9 [44] and R statistical software version 4.2.0 (R Foundation for Statistical Computing, Vienna, Austria) [45].

## 5. Conclusions

The therapeutic index of anticancer drugs is typically extremely limited, making it crucial to administer the correct dosages to ensure that the patient receives the maximal benefits without being at risk of life-threatening toxicities. The extensive use of VCR in treating children’s cancer is because of its high effectiveness; however, VCR treatment may result in VIPN. VIPN is dose-limiting and may compromise the effectiveness of treatment or lead, in the most severe cases, to permanent disability and significantly affect the quality of life of cancer survivors. Clinical practice demonstrates that VCR patients can experience VIPN with varying degrees of commitment and severity. This suggests that individuals differ in their response to antineoplastic treatment as well as their susceptibility to drug-related adverse effects. Inheritance of specific variations in the genes that code for the drug’s transporters and target proteins can help to explain and predict VIPN. Pharmacogenetics has emerged as a tool to anticipate individual responses to drugs, enabling personalized therapeutic management

Our results confirmed that age at diagnosis and the G allele of the *ABCC1* rs246240 SNP are significantly associated with VIPN in pediatric cancer patients. These associations remained significant after adjusting for multiple comparisons. Future research should explore the underlying biological mechanisms of these associations through additional and longitudinal studies, investigate strategies to mitigate the risk of VIPN and expand study cohorts to validate the generalizability of the findings and tailor treatment protocols according to the patients’ genetic profiles.

## Figures and Tables

**Table 1:** Sociodemographic and clinicopathologic characteristics of 88 pediatric cancer patients treated with VCR.

Variable	*n*	%	Median (p25–p75)
Sex			
Female	42	47.73	
Male	46	52.27	
Family history of cancer			
Yes	45	51.14	
No	43	48.86	
Disease			
Acute lymphocytic leukemia	49	55.68	
Non-Hodgkin lymphoma	15	17.05	
Ewing sarcoma	7	7.95	
Hodgkin lymphoma	6	6.82	
Ependymoma	4	4.55	
Other cancers	7	7.95	
Age at diagnosis			6 (3–9)
Neurotoxicity			
Yes	10	11.36	
No	78	88.64	
Peripheral Neuropathy			
Yes	8	9.09	
No	80	90.91	

Qualitative variables: n: frequency; %: percentage. Quantitative variables: non-normal distribution: median (percentiles: p25–p75).

**Table 2:** Clinical variables and peripheral neuropathy.

Characteristics	N	Peripheral Neuropathy		*p*-Value	OR	95% CI
		No *n* (%)	Yes *n* (%)			
Gender						
Female	42	36 (85.7)	6 (14.3)	0.144 *		
Male	46	44 (95.7)	2 (4.3)			
Family history of cancer						
No	43	39 (90.7)	4 (9.3)	0.951 *		
Yes	45	41 (91.1)	4 (8.9)			
Disease						
Acute lymphocytic leukemia	49	46 (93.9)	3 (6.1)	0.051 *	1	
Non-Hodgkin lymphoma	15	14 (93.3)	1 (6.7)		1.10	0.05–9.35
Other cancers	7	7 (100.0)	0 (0.0)		1.30 × 10^−7^	NA-9.49 × 10^139^
Ewing sarcoma	7	6 (85.7)	1 (14.3)		2.56	0.12–24.08
Hodgkin lymphoma	6	3 (50.0)	3 (50.0)		15.33	2.11–126.49
Ependymoma	4	4 (100.0)	0 (0.0)		1.30 × 10^−7^	NA-1.60 × 10^187^
Age at diagnosis	88	6 (3.0–8.0)	9 (7.0–13.25)	0.029	1.22	1.03–1.50

N: frequency; %: percentage; OR: odds ratio; CI: confidence interval. * *p*-value for the Fisher’s test.

**Table 3:** Genes and peripheral neuropathy.

Gene	SNP	Genotype	N	Peripheral Neuropathy		*p*-Value	Ref	OR	95% CI
				No *n* (%)	Yes *n* (%)				
*ABCB1*	rs1128503	AA	23	21 (91.3)	2 (8.7)	0.575 *			
		AG	39	34 (87.2)	5 (12.8)				
		GG	26	25 (96.2)	1 (3.8)				
		A	62	55 (88.7)	7 (11.3)	0.427 *			
		G	65	59 (90.8)	6 (9.2)	1 *			
*ABCC1*	rs246240	AA	68	65 (95.6)	3 (4.4)	0.004 *	AA	1	
		AG	19	15 (78.9)	4 (21.1)			5.78	1.16–32.03
		GG	1	0 (0.0)	1 (100.0)			3.39 × 10^8^	2.37–NA
		A	87	80 (92.0)	7 (8.0)	0.090 *			
		G	20	15 (75.0)	5 (25.0)	0.013 *	AA	7.00	1.21–50.19
*ABCC2*	rs717620	CC	45	41 (91.1)	4 (8.9)	0.669 *			
		CT	33	29 (87.9)	4 (12.1)				
		TT	10	10 (100.0)	(0.0)				
		C	78	70 (89.7)	8 (10.3)	0.589 *			
		T	43	39 (90.7)	4 (9.3)	1 *			
*CEP72*	rs924607	CC	28	26 (92.9)	2 (7.1)	0.883 *			
		CT	46	41 (89.1)	5 (10.9)				
		TT	14	13 (92.9)	1 (7.1)				
		C	74	67 (90.5)	7 (9.5)	1 *			
		T	60	54 (90.0)	6 (10.0)	1 *			

N: frequency; %: percentage; OR: odds ratio; CI: confidence interval; SNP: single nucleotide polymorphism. * *p*-value for the Fisher’s test.

**Table 4:** Multivariate regression analysis for peripheral neuropathy according to sociodemographic, clinicopathologic characteristics and gene polymorphisms.

Characteristics	Peripheral Neuropathy *			
	*p*-Value	q-Value	OR	95% CI
*ABCC1* rs246240 (G vs. AA)	0.006	0.025	12.45	2.26–100.42
Age at diagnosis	0.016	0.050	1.33	1.07–1.75

* Any degree. OR: odds ratio; CI: confidence interval. Model *p*-value < 0.001.

**Table 5:** Gene polymorphisms and TaqMan IDs.

Gene	SNP	dbSNP ID	Assay ID
*ABCB1*	A > G	rs1128503	C___7586662_10
*ABCC1*	A > G	rs246240	C___1003698_10
*ABCC2*	C > T	rs717620	C___2814642_10
*CEP72*	C > T	rs924607	C___8292459_20

Db: database; ID: identification; SNP: single nucleotide polymorphism.

## Data Availability

Data is contained within the article and Supplementary Materials.

## Supplementary Materials

The following supporting information can be downloaded at: https://www.mdpi.com/article/10.3390/ijms25168797/s1.

## Abbreviations

ALL, acute lymphocytic leukemia; ABC, ATP-binding cassette; *CEP72*, centrosomal protein 72; CI, confidence intervals; D’, Lewontin’s D prime; HWE, Hardy–Weinberg equilibrium; HUVN, Virgen de las Nieves University Hospital; ID: identification; LD, linkage disequilibrium; MAF, minor allele frequency, MRP1, Multidrug resistance-associated protein 1; NCI-CTCAE, National Cancer Institute—Common Terminology Criteria for Adverse Events; OR, odds ratios; PN, peripheral neuropathy; PCR, polymerase chain reaction; r^2^, LD coefficient; SNP, single nucleotide polymorphism; VCR, vincristine; VIPN, vincristine-induced peripheral neuropathy.
