# Supplementary material for: Precision Medicine in Childhood Cancer: The Influence of Genetic Polymorphisms on Vincristine-Induced Peripheral Neuropathy
Source: Int J Mol Sci. 2024 Aug 13;25(16):8797. doi: 10.3390/ijms25168797 (PMC11354794; doi:10.3390/ijms25168797)
Supplement: Supplementary file 1 [file ijms-25-08797-s001.zip › Table S1. Hardy-Weinberg equilibrium for the SNPs included in the study.pdf]

Table S1. *Hardy-Weinberg equilibrium* for the SNPs included in the study.

Chr	SNP	Gene	Minor Allele	Major Allele	Genotype counts	Observed heterozygosity	Expected heterozygosity	p-value
5	rs924607	CEP72	T	C	14/46/28	0.5227	0.4873	0.6613
7	rs1128503	ABCB1	A	G	23/39/26	0.4432	0.4994	0.2914
10	rs717620	AABCC2	T	C	10/33/45	0.375	0.4209	0.3147
16	rs246240	ABCC1	G	A	1/19/68	0.2159	0.2102	1
