# Supplementary material for: Precision Medicine in Childhood Cancer: The Influence of Genetic Polymorphisms on Vincristine-Induced Peripheral Neuropathy
Source: Int J Mol Sci. 2024 Aug 13;25(16):8797. doi: 10.3390/ijms25168797 (PMC11354794; doi:10.3390/ijms25168797)
Supplement: Supplementary file 1 [file ijms-25-08797-s001.zip › Table S2 Minor allele frequencies for the SNPs studied.pdf]

Table S2 Minor allele frequencies for the SNPs studied.

Chr	SNP	Gene	Minor Allele	Major Alelle	MAF
5	rs924607	CEP72	T	C	0.4205
7	rs1128503	ABCB1	A	G	0.483
10	rs717620	ABCC2	T	C	0.3011
16	rs246240	ABCC1	G	A	0.1193
